# Development and validation of a prediction model for the early occurrence of acute kidney injury in patients with acute pancreatitis

**DOI:** 10.1080/0886022X.2023.2194436

**Published:** 2023-03-30

**Authors:** Simin Wu, Qin Zhou, Yang Cai, Xiangjie Duan

**Affiliations:** a Department of Respiratory Medicine, The First Affiliated Hospital of Yangtze University, Jingzhou, P.R. China; b Department of Intensive care Medicine, The First People’s Hospital of Changde, Changde, P.R. China; c Department of Infectious Diseases, The First People’s Hospital of Changde, Changde, P.R. China

**Keywords:** Acute pancreatitis, acute kidney injury, nomogram, MIMIC, risk factors

## Abstract

**Background:**

Acute pancreatitis (AP) is associated with a high incidence of acute kidney injury (AKI). This study aimed to develop a nomogram for predicting the early onset of AKI in AP patients admitted to the intensive care unit.

**Method:**

Clinical data for 799 patients diagnosed with AP were extracted from the Medical Information Mart for Intensive Care IV database. Eligible AP patients were randomly divided into training and validation cohorts. The independent prognostic factors for the early development of AKI in AP patients were determined using the all-subsets regression method and multivariate logistic regression. A nomogram was constructed for predicting the early occurrence of AKI in AP patients. The performance of the nomogram was evaluated based on the area under the receiver operating characteristic curve (AUC), calibration curves and decision curve analysis (DCA).

**Results:**

Seven independent prognostic factors were identified as predictive factors for early onset AKI in AP patients. The AUC of the nomogram in the training and validation cohorts were 0.795 (95% CI, 0.758–0.832) and 0.772 (95% CI, 0.711–0.832), respectively. The AUC of the nomogram was higher compared with that of the BISAP, Ranson, APACHE II scores. Further, the calibration curve revealed that the predicted outcome was in agreement with the actual observations. Finally, the DCA curves showed that the nomogram had a good clinical applicability value.

**Conclusion:**

The constructed nomogram showed a good predictive ability for the early occurrence of AKI in AP patients.

## Introduction

Acute pancreatitis (AP) is a common disease of sudden onset. The disease has a diverse clinical course that ranges from mild, to moderate to severe acute pancreatitis (SAP), with a high mortality rate of 36–50% [1]. The pathophysiological mechanisms of AP are not well understood. However, pancreatic enzymes and various inflammatory mediators are thought to contribute to systemic inflammatory response syndrome and multi-organ failure [2]. In some patients, AP may lead to acute kidney injury (AKI). AKI in AP has an incidence rate of 10–42% and a high mortality rate of about 80% [3,4]. Therefore, an early and accurate diagnosis of AKI in AP may help improve the disease prognosis. A few studies have explored factors and establish predictive models for AKI in AP patients. However, these studies had small sample sizes and lacked the accuracy of the predictive models [5–8]. Early and accurate diagnosis of AKI in AP patients has remained challenging in clinical practice. Therefore, this study aimed to identify the prognostic factors for AKI in AP patients from a large database. In addition, we established and validated an easy-to-use prognostic nomogram, to help clinicians to prevent and treat AKI.

## Methods

### Data source

AP patients were obtained from the Medical Information Mart for Intensive Care-IV 1.0 (MIMIC-IV v1.0) database [9]. The MIMIC-IV is a large, single-center, open-access database, containing data from 382,278 patients with 524,740 admissions to Beth Israel Deaconess Medical Center in Boston from 2008 to 2019. Relevant data, including patient demographics, hourly vital signs, laboratory microbial culture and imaging test results, surgical procedures, medication records, and survival data, were collected.

The use of data from the MIMIC-IV database was approved by the Institutional Review Board of the Beth Israel Deaconess Medical Center and Massachusetts Institute of Technology. Since the data were deidentified, there was no requirement for informed consent. To access the database, we first completed the required online courses and an examination (Record ID: 42039823).

### Patients and data variables

Data were extracted using Structured Query Language (SQL) programming in PostgreSQL (version 14.0), and the SQL script codes used to extract patients' information were obtained from the GitHub website (https://github.com/MIT-LCP/mimic-code/tree/main/mimic-iv) [10].

Using the International Classification of Diseases (ICD), ninth revision (ICD-9, code 577.0) and tenth revision (ICD-10, code K85%), we identified the patients diagnosed with AP from the MIMIC-IV v1.0 database. The exclusion criteria were age less than 18 years old and intensive care unit (ICU) stay < 24 h and patients with a history of chronic renal failure, including a previous history of a kidney transplant. For patients who had multiple admissions to the ICU, data were only obtained from the first admission.

After identifying eligible patients, we collected AP patient’s baseline parameters immediately after admission to the ICU, including demographic information, previous medical history, vital signs, laboratory indicators, injury factors, interventions, and disease severity scores. The vital signs and laboratory indicators are the first value within the first 24 h after ICU, and intervention measures and the severity scores of the disease are evaluated within the first 24 h after ICU.

The outcome measure was determined as the development of AKI within seven days of admission to the ICU. A diagnosis of AKI was based on the 2012 Kidney Disease: Improving Global Outcomes (KIDGO) guidelines as follows: elevated serum creatinine (SCr) after 48 h by ≥26.5 μmol/L (0.3 mg/dl); or ≥50% of the baseline SCr value (increased by 1.5 times), or a urine output of more than six hours of <0.5 mL/kg/h [11]. Baseline SCr is defined as the lowest SCr in the past 7 days.

### Development and validation of the prediction model

Missing data were common in the MIMIC database. In this study, variables with a deletion rate greater than 20% were excluded. The extracted variables and missing proportions of variables are shown in Appendix 1. The trimming method is used to deal with abnormal values, and the technique of multiple imputations is used to fill in missing data [12].

The patients were randomly assigned to the training cohort (70%) and validation cohort (30%). Subsequently, a nomogram was established based on the training cohort and validated in the validation cohort.

### Statistical analysis

Measurement data conforming to the normal distribution and homogeneity of variance were expressed as the mean ± standard deviation. Data between groups were compared using the independent t-test. The unequal variance *t*-test was used to compare data between groups with unequal variances. Data with skewed distribution were expressed as median and quartiles and were compared using the Mann–Whitney *U* test. Furthermore, categorical variables were presented with count (%) and were compared using the *χ^2^* test.

Due to a large number of variables, the independent prognostic factors were filtered through two steps. First, a preliminary screening was conducted to identify possible predictors using the all-subsets regression method to avoid overfitting and increase the degree of the model. Subsequently, multivariate logistic regression was carried out. A nomogram was then constructed using all the independent prognostic factors.

The nomogram was validated using multiple metrics. The discriminative ability of the nomogram was evaluated using the area under the receiver operating characteristic curve (AUC). The obtained AUC values were compared with AUC values of the bedside index for the severity in acute pancreatitis (BISAP) scores, Ranson scores, and acute physiology and chronic health evaluation (APACHE) II scores. Further, a calibration curve was plotted to evaluate the calibrating ability of the nomogram. Moreover, Decision curve analysis (DCA) was used to assess the clinical significance of the nomogram. A *p*-value <0.05 was considered statistically significant. Data were analyzed using the Stata software (version 15.1) and R software (version 4.1.0), including tidyverse, mice, caret, leaps, glmnet, rms, pROC, and ggDCA.

## Results

### Baseline characteristics of the study cohort

**Figure 1.**
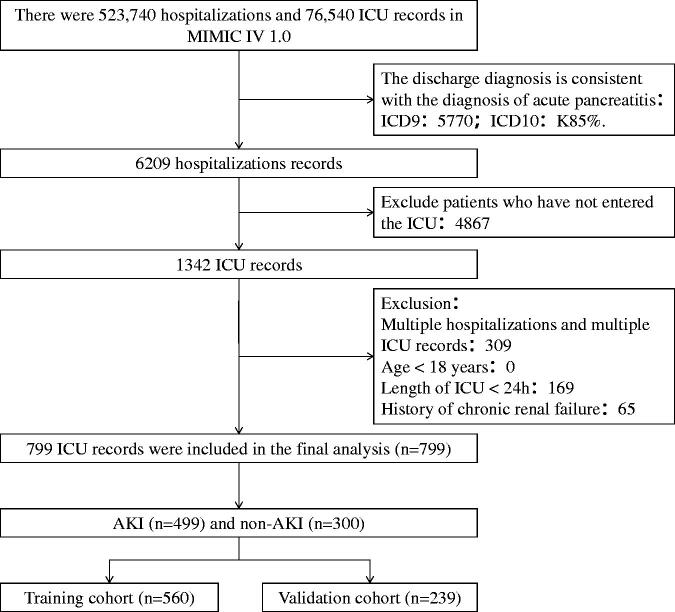
A flow chart showing the screening criteria for acute pancreatitis.

A total of 799 patients with acute pancreatitis were identified from the MIMIC-IV database. The patients were randomly assigned to the training cohort (*n* = 560) and the validation cohort (*n* = 239). Patients were screened for acute pancreatitis as shown in Figure 1. Further, the AP patients were divided into two groups (AKI and non-AKI groups) based on whether the patients developed AKI within seven days of admission into the ICU. The overall incidence rate of AKI was 62.45% (499/799). Further analysis revealed that 12.52% (100/799), 26.53% (212/799), and 23.40% (187/799) had stage 1, stage 2, and stage 3 AKI, respectively (Appendix 2).

Clinical data of patients in the AKI group were compared to that of patients in the non-AKI group. The results revealed that patients in the AKI group were older, with higher mortality rates and APACHE II, BISAP, and Ranson scores compared with the non-AKI group. In addition, patients in the AKI group were more likely to require mechanical ventilation and renal replacement therapy, use vasoactive drugs, and had longer ICU and hospital stays than those in the non-AKI group. Differences between the AKI and non-AKI groups were statistically significant (all *p*-values <0.05). See Table 1 for details.

**Table 1.** A comparison of the baseline characteristics between the AKI and non-AKI groups.

Variables	Non-AKI group (*n* = 214)	AKI group (*n* = 346)	Statistics (U/*χ*^2^)	*p* Value
Age, (years)	55.12 (17.79)	61.68 (16.74)	*t* = −4.399	<0.001
Male, *n*(%)	92 (42.99%)	131 (37.86%)	*χ*^2^ = 1.452	0.228
Ethnicity, *n*(%)			*χ*^2^ = 12.780	0.002
White	121 (56.54%)	239 (69.08%)		
Black	29 (13.55%)	21 (6.07%)		
Other	64 (29.91%)	86 (24.86%)		
Comorbid disease, *n*(%)				
Hypertension	131 (61.21%)	229 (66.18%)	*χ*^2^ = 1.423	0.233
Diabetes	64 (29.91%)	116 (33.53%)	*χ*^2^ = 0.794	0.373
Myocardial infarction	15 (7.01%)	37 (10.69%)	*χ*^2^ = 2.131	0.144
Congestive heart failure	26 (12.15%)	82 (23.70%)	*χ*^2^ = 11.331	0.001
Atrial fibrillation	34 (15.89%)	83 (23.99%)	*χ*^2^ = 5.250	0.022
Hyperlipidemia	81 (37.85%)	137 (39.60%)	*χ*^2^ = 0.169	0.681
Chronic lung disease	42 (19.63%)	87 (25.14%)	*χ*^2^ = 2.271	0.132
CKD	17 (7.94%)	45 (13.01%)	*χ*^2^ = 3.441	0.064
Liver disease	54 (25.23%)	111 (32.08%)	*χ*^2^ = 2.983	0.084
Malignant tumor	15 (7.01%)	34 (9.83%)	*χ*^2^ = 1.314	0.252
Obesity	20 (9.35%)	51 (14.74%)	*χ*^2^ = 3.475	0.062
History of drinking alcohol	86 (40.19%)	125 (36.13%)	*χ*^2^ = 0.928	0.335
Vital signs				
Heart rate, (times/min)	101.48 (21.33)	101.89 (21.15)	*t*= −0.222	0.825
Respiratory, (times/min)	20.80 (6.32)	22.27 (6.56)	*t*=–2.382	0.018
Temperature, (°C)	37.01 (0.83)	36.82 (0.92)	*t* = 2.444	0.015
Systolic blood pressure, (mmHg)	133.57 (23.25)	125.34 (25.27)	*t* = 3.849	<0.001
Diastolic blood pressure, (mmHg)	77.94 (17.88)	72.50 (19.29)	*t* = 3.373	0.001
Mean arterial pressure, (mmHg)	90.92 (17.51)	85.10 (19.75)	*t* = 3.556	<0.001
Oxygen saturation, (%)	96.30 (3.15)	95.66 (3.73)	*t =* 2.099	0.036
Laboratory indicators, (Reference range, Unit)				
WBC, (4–10, ×10^9^/L)	12.00 (8.40–17.28)	13.60 (9.53–19.10)	*U* = 32218	0.010
HB, (14–18, g/dL)	11.53 (2.34)	11.61 (2.76)	*t*= −0.400	0.689
HCT, (36–48, %)	34.61 (6.85)	35.22 (8.18)	*t*= −0.957	0.339
PLT, (150–440, ×10^9^/L)	203.50 (146.75–294.00)	202.00 (138.00–288.50)	*U* = 36242	0.675
RDW, (10.5–15.5, %)	14.70 (1.82)	15.18 (2.03)	*t*= −2.895	0.004
ALT, (0–40, IU/L)	47.00 (22.00–124.25)	56.00 (26.00–167.00)	*U* = 34191	0.128
AST, (0–40, IU/L)	62.00 (32.00–131.50)	85.50 (39.25–202.75)	*U* = 31845	0.005
ALP, (35–105, IU/L)	104.50 (68.25–162.75)	108.00 (71.00–173.50)	*U* = 35973	0.573
Total bilirubin, (0–1.5, mg/dL)	0.90 (0.50–2.30)	1.20 (0.60–3.40)	*t*= −4.636	<0.001
AG, (10–18, mEq/L)	15.00 (13.00–19.00)	16.00 (13.00–20.00)	*U* = 34415	0.160
Bicarbonate, (22–32, mEq/L)	22.00 (19.00–25.00)	21.00 (17.00–24.75)	*U* = 32514	0.015
BUN, (6–20, mg/dL)	15.00 (9.25–26.75)	21.50 (14.00–38.00)	*U* = 26776	<0.001
SCr, (0.5–1.2, mg/dL)	0.90 (0.60–1.40)	1.10 (0.80–1.98)	*U* = 28180	<0.001
Serum Sodium, (133–145, mg/dL)	137.67 (5.63)	137.92 (5.80)	*t*= −0.487	0.626
Serum Chlorine, (96–108, mg/dL)	102.73 (7.65)	103.53 (7.49)	*t*= −1.361	0.174
Serum Potassium, (3.3–5.1, mg/dL)	4.20 (0.92)	4.24 (0.87)	*t*= −0.575	0.566
Serum Calcium, (8.4–10.3, mg/dL)	8.05 (1.10)	7.84 (1.07)	*t* = 2.261	0.024
Blood glucose, (70–100, mg/dL)	123.00 (103.00–178.00)	130.00 (104.00–178.75)	*U* = 36049	0.601
INR, (0.9–1.1)	1.20 (1.10–1.40)	1.30 (1.20–1.60)	*U* = 28485	<0.001
PT, (9.4–12.5, s)	15.05 (6.50)	17.25 (8.07)	*t*= −3.641	<0.001
APTT, (25–36.5, s)	30.27 (7.97)	37.13 (20.73)	*t*= −5.292	<0.001
Interventions, *n*(%)				
Mechanical ventilation	43 (20.09%)	175 (50.58%)	*χ^2^* = 51.684	<0.001
RRT	2 (0.93%)	33 (9.54%)	*χ^2^* = 16.701	<0.001
Vasoactive drugs are used	21 (9.81%)	131 (37.86%)	*χ*^2^ = 52.600	<0.001
Injury factors, *n*(%)				
Sepsis	96 (44.86%)	274 (79.19%)	*χ*^2^ = 69.517	<0.001
Antibiotics are used	117 (54.67%)	255 (73.70%)	*χ*^2^ = 21.463	<0.001
Disease severity score, (points)				
CCI score	3.00 (2.00–5.00)	5.00 (3.00–7.00)	*U* = 26948	<0.001
BISAP score	2.00 (2.00–3.00)	3.00 (2.00–4.00)	*U* = 26794	<0.001
Ranson score	2.00 (2.00–3.00)	3.00 (2.00–5.00)	*U* = 21755	<0.001
APACHE II score	16.59 (6.73)	23.11 (7.73)	*t*= −10.168	<0.001
Outcome-related measures				
Length of ICU, (days)	2.02 (1.35–3.35)	4.51 (2.19–11.86)	*U* = 19278	<0.001
Length of hospital, (days)	8.27 (4.71–15.06)	15.39 (7.88–26.17)	*U* = 24830	<0.001
ICU mortality, *n*(%)	1 (0.47%)	39 (11.27%)	*χ*^2^ = 23.271	<0.001
Hospital mortality, *n*(%)	7 (3.27%)	55 (15.90%)	*χ*^2^ = 21.405	<0.001
AKI stage, *n*(%)				/
AKI stage 1	0 (0.00%)	64 (18.50%)		
AKI stage 2	0 (0.00%)	151 (43.64%)		
AKI stage 3	0 (0.00%)	131 (37.86%)		

AKI refers to acute kidney injury, CKD refers to chronic kidney disease, WBC refers to white blood cell count, HB refers to hemoglobin, HCT refers to hematocrit, PLT refers to platelets, RDW refers to red cell volume distribution width, ALT refers to alanine aminotransferase, AST refers to aspartate aminotransferase, ALP refers to alkaline phosphatase, AG refers to anion gap, BUN refers to blood urea nitrogen, SCr refers to serum creatinine, INR refers to international normalized ratio, PT refers to prothrombin time, APTT refers to activating partial thrombin time, RRT refers to renal replacement therapy, CCI refers to Charson comorbidity index, BISAP refers to bedside index for the severity in acute pancreatitis, APACHE II refers to acute physiology and chronic health evaluation II, ICU refers to intensive care unit; 1 kPa ≈ 7.5 mmHg.

### Early potential predictors for AKI in AP patients

**Figure 2.**
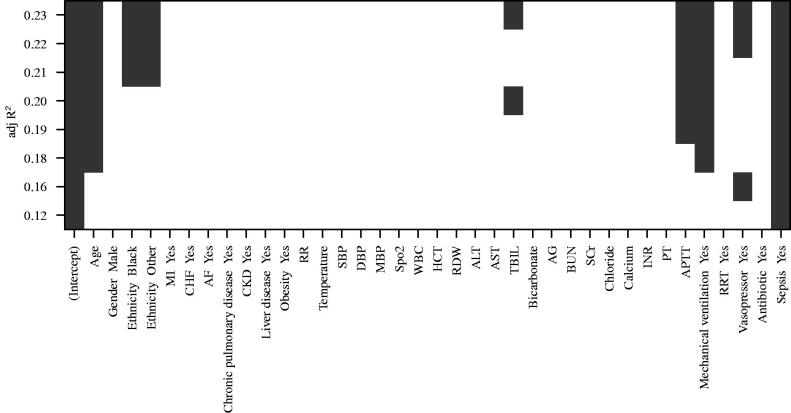
Potential predictors for AKI in AP patients.

Variables in the training cohort with a *p*-value <0.2 between the AKI and non-AKI groups were selected as potential predictors for AKI. The variables were screened using the all-subsets regression model. Consequently, seven variables were identified, including age, race, total bilirubin, activated partial thromboplastin time (APTT), need for mechanical ventilation, use of vasoactive drugs, and sepsis as the potential predictors of AKI in AP, after adjusting for the maximum value of *R*^2^ (adjR^2^). See Figure 2 for details.

### Construction of the nomogram for predicting the risk of developing AKI

Multivariate logistic regression was performed on the seven variables identified in the all-subsets regression. The results showed that age, ethnicity, total bilirubin, activated partial thromboplastin time, need for mechanical ventilation, use of vasoactive drugs, and sepsis were all independent risk factors for early development of AKI in AP patients as shown in Table 2.

**Table 2.** Results of the multivariate logistic regression analysis showing predictors for the early occurrence of AKI in AP Patients.

Variables	Multivariate logistic regression		
	*β* value	OR (95%CI)	*p* Value
Age, (years)	0.018	1.018 (1.006–1.030)	0.003
Ethnicity, *n*(%)			
White		Ref	
Black	−1.128	0.324 (0.152–0.669)	0.003
Other	−0.839	0.432 (0.268–0.692)	<0.001
Total bilirubin, (mg/dL)	0.086	1.090 (1.025–1.173)	0.012
APTT, (s)	0.032	1.032 (1.013–1.058)	0.005
Mechanical ventilation, *n*(%)	0.912	2.489 (1.517–4.135)	<0.001
Use of vasoactive drugs, *n*(%)	0.950	2.586 (1.428–4.820)	0.002
Sepsis, *n*(%)	0.802	2.231 (1.430–3.483)	<0.001

APTT: refers to activating partial thrombin time; OR: refers to odds ratio; CI: refers to confidence interval.

**Figure 3.**
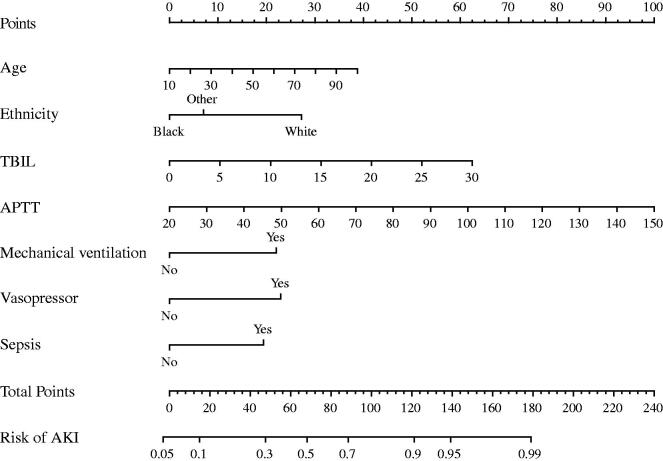
A nomogram based on age, ethnicity, total bilirubin, activated partial thromboplastin time, need for mechanical ventilation, use of vasoactive drugs, and sepsis.

Further, a nomogram was constructed based on the results of the multivariate logistic regression. The risk scores for each factor included in the nomogram are shown in Figure 3. The higher the score, the higher the risk for AP patients to develop AKI.

### Evaluation of the effectiveness of the nomogram

**Figure 4.**
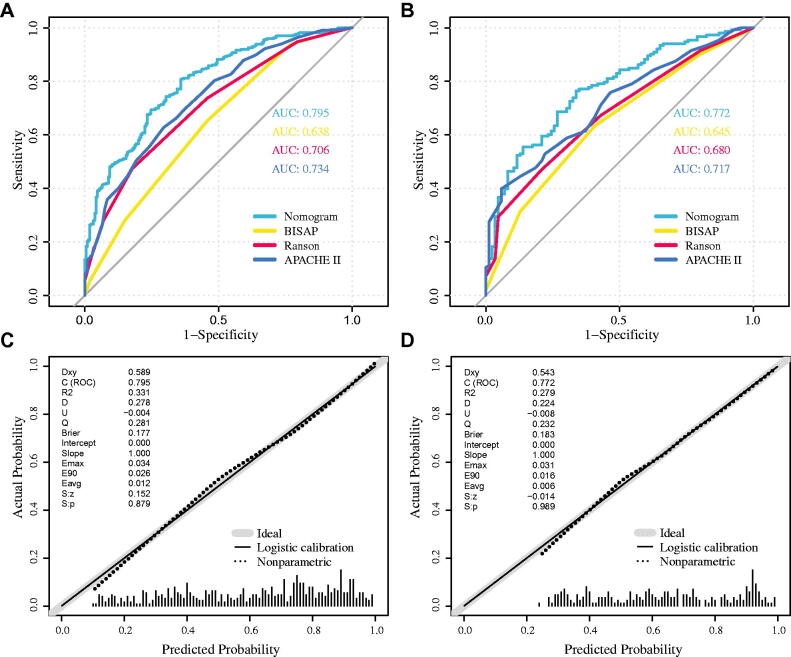
(A,B) ROC curves of the nomogram, BISAP score, Ranson score, and APACHE II score for predicting the likelihood of developing AKI in AP patients. (A) is the training cohort, (B) is the validation cohort. (C,D) Calibrate curves of the nomogram for predicting AKI in AP patients; (C) is the training cohort, (D) is the validation cohort.

In the training cohort, the AUC value was 0.795 (95% CI, 0.758–0.832). Further, the BISAP, Ranson, and APACHE II scores showed AUC values of 0.638(95% CI, 0.593–0.68), 0.706(95% CI, 0.664–0.748), and 0.734(95% CI, 0.693–0.776), respectively. The differences were statistically significant (*D* = 5.217, 3.094, 2.109, *p* < 0.05). The validation cohort had AUC values of 0.772 (95% CI, 0.711–0.832). In addition, the BISAP, Ranson, and APACHE II showed AUC values of 0.645(95% CI, 0.576–0.714), 0.680(95% CI, 0.613–0.746), and 0.717(95% CI, 0.652–0.781), respectively. The difference was not statistically significant (*D* = 2.715, *d* = 2.017, *d* = 1.227, *p* = 0.007, *p* = 0.044, *p* = 0.221). Figure 4 shows calibration curves for the nomogram, the training cohort, and the validation cohort. The curve is close to the front diagonal, indicating that the nomogram had good prediction performance.

### Clinical use of the nomogram

**Figure 5.**
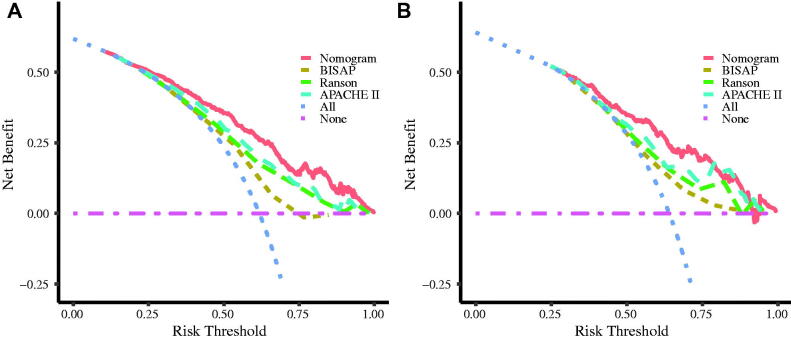
DCA of the nomogram, BISAP score, Ranson score, and APACHE II score predict the early occurrence of AKI in AP patients; A is the training cohort, and B is the validation cohort. Note: The pink dotted line (None) represents the net benefit rate in the non-AKI group, while the purple dotted line (All) represents the net benefit rate of the AKI group.

DCA was plotted with net benefit as the ordinate coordinate and high-risk threshold probability as the abscissa. The results showed that the nomogram had a good clinical value. The DCA revealed that patients had a higher net benefit than BISAP, Ranson, and APACHE II scores, as shown in Figure 5.

## Discussion

AKI is a common complication of AP patients. AKI was independently associated with a higher mortality rate in AP patients [13]. Therefore, early prediction of the risk of developing AKI in acute pancreatitis may help lower the mortality rate of the disease. The MIMIC database contains a large amount of data on the clinical diagnosis and treatment of critically ill patients, thus providing data for scientific research [14]. In our study, the risk factors of early AKI in AP patients were comprehensively screened using the all-subsets regression method and multivariate logistic regression. The results indicated that age, ethnicity, Total bilirubin (TBIL), activating partial thrombin time (APTT), mechanical ventilation, vasopressor and sepsis were independent risk factors for early AKI in AP patients, which was consistent with previous studies. The constructed nomogram had a good predicting ability, was based on a large sample size obtained from the MIMIC database, and had a good clinical utility.

An increase in age is associated with a decline in renal function. Patients with acute pancreatitis have a higher incidence rate of developing AKI associated with deterioration of physiological functioning with age [15]. In addition, age is a known predictor of AP severity and mortality [16]. For example, older patients have a higher risk of developing systemic complications, such as multiple organ failure [15]. The risk of developing kidney diseases varies amongst different ethnicities. For example, African Americans have a 2–4 times higher risk of developing chronic kidney disease or end-stage renal disease than Caucasians. In addition, African Americans have a higher risk of developing AKI than Caucasians. These differences may be attributed to differences in economic conditions, social status, and genetics between African Americans and Caucasians. However, the present study revealed that Caucasians with acute pancreatitis were more likely to develop AKI than African Americans, possibly due to their diet and a higher basal metabolic rate [17].

Furthermore, the present study identified hyperbilirubinemia to be an independent risk factor for the early development of AKI in AP patients. Hyperbilirubinemia may induce the formation of reactive oxygen species in mitochondria, damaging the tubular epithelial cells and exacerbating ischemic reperfusion kidney injury [18,19]. Bilirubin is a potent antioxidant and has been shown to be. An elevated bilirubin level is an independent risk factor for the development of AKI given that the pathogenesis of AP is closely related to oxidative stress [20–22].

Coagulation dysfunction is an independent risk factor for AKI [23]. In addition, a prolonged APTT has a predictive value in sepsis-associated AKI [24,25]. The present study revealed that an APTT value greater than 20 was associated with a poorer prognosis. According to Liu et al. differences in APTT values and thrombin time (TT) values were risk factors for organ failure in AP patients [26], consistent with the present study.

The analysis revealed that mechanical ventilation (MV) predicted AKI in AP, which was consistent with findings from a previous study by Shi et al. [27]. Studies have shown that acute respiratory failure due to acute pancreatitis necessitates the use of MV in patients admitted to the ICU. The use of MV can lead to acute lung injury, thus worsening hypoxia, causing vasoconstriction, decreased renal perfusion, and a decreased glomerular filtration rate. In addition, MV increases intrathoracic pressure, lower venous return and the mean arterial pressure, which may cause prerenal hypoperfusion and lead to acute renal injury [28,29]. This study demonstrated that vasoactive drugs can predict AKI in AP. Consistent with this finding, a previous study revealed that the need for MV, the use of vasopressor agents, and RRT were risk factors for higher mortality in AP patients [30]. Critically ill patients require higher doses of vasopressor agents to help regulate blood pressure.

Up to one-third of patients with necrotizing pancreatitis develop necrotic infection [31]. The incidence of SAP is biphasic and is closely related to the early and persistent presence of organ or multi-organ dysfunction in the first week of the disease course. Clinical sepsis caused by multiple organ failure syndrome due to infectious necrosis occurs later in the first week [32]. A prospective study revealed that the most important independent predictors of AP mortality were persistent organ failure and infectious pancreatic necrosis complicated by multidrug-resistant organisms [33]. The extent of necrosis has been correlated with organ failure and mortality, this may be associated with further complications of the pancreas and extrapancreatic necrosis, which could predispose the patient to infections, pseudoaneurysms, and intestinal fistulas. Sepsis is also an independent risk factor for acute kidney injury [34]. In addition, unsaturated fatty acids could worsen systemic inflammation and organ failure. The substances released by the necrotizing pancreas are involved in the pathogenesis of AKI [35]. The main and secondary mediators of systemic inflammatory response play a key role in the pathogenesis of AP and significantly participate in the development of AP-AKI and other organ dysfunction [2]. TNF-α, released during the happens of AP, interacts with ductal pancreatic cells, glomeruli and renal tubules, accompanied by obvious local inflammatory reaction cells, continuous tissue ischemia, interstitial edema and cell necrosis.

Currently, the APACHE II, BISAP, and Ranson scores are clinically used to predict the prognosis of AP patients in the ICU [36–38]. there is no relevant research to calculate the predictive value of the above score for early AKI in patients with acute pancreatitis in the ICU. The nomogram showed a good degree of differentiation and calibration in the training and validation cohorts. The AUC of the APACHE II, BISAP, and Ranson scores in the training and validation cohorts were lower than that of the nomogram. In addition to validating the APACHE II score in the validation cohort (This may be related to the sample size of the validation set, and further sample size verification is required in the future), the AUC between the nomogram and the scores showed statistically significant differences. Compared with the above scoring system, the variables of the nomogram can be simply obtained and are easy to calculate. Further, the nomogram can be used to stratify the risk of AKI in AP patients admitted to the ICU, thus guiding physicians to offer targeted management.

However, this study had some limitations. First, this study was retrospective, and single-centered. Therefore, future studies should be conducted prospectively in multiple centres to improve clinical utility and external validation, respectively. In addition, the nomogram did not include novel biomarkers or imaging results, which could hinder the performance of the model. The model did not include other factors, such as causes of acute pancreatitis, classification of acute pancreatitis, intra-abdominal hypertension and abdominal compartment syndrome that could potentially affect the development of AKI in AP. Third, the sample size of this study is not large, and only internal verification is used to evaluate the accuracy and effectiveness of the model. Future studies should employ large sample sizes, and include more variables to validate our findings.

## Data Availability

The data that support the findings of this study are openly available on the MIMIC-IV website at https://physionet.org/content/mimiciv/1.0/.

## Appendix 1.

Data extraction variables and deletion ratios of patients with acute pancreatitis in MIMIC-IV 1.0 database

**Table:**

Variables	Missing proportions	Variables	Missing proportions
Patient code			
subject_id	0%	hadm_id	0%
stay_id	0%	icd_code	0%
icd_version	0%	seq_num	0%
Demographic information and hospitalization information			
Age	0%	Sex	0%
Ethnicity	0%	Height	43.50%
Weight	2.80%	Time of death	0%
Admission time	0%	Discharge time	0%
Time into the ICU	0%	Out of the ICU time	0%
Length of hospital stay	0%	Length of ICU hospital stay	0%
Hospitalized death	0%	ICU death	0%
First hospital stay for the subject	0%	First ICU stay for the current hospitalization	0%
Vital signs (first value within the first 24 h of admission to the ICU)			
Heart rate	0%	Systolic blood pressure	0.20%
Respiratory	0.30%	Diastolic blood pressure	0.20%
Temperature	0.30%	Mean arterial pressure	0.10%
Oxygen saturation	0%		
Laboratory indicators (first value within the first 24 h of admission to the ICU)			
White blood cell	0.40%	Hemoglobin	0.30%
Hemoglobin	0.40%	Hematocrit	0.20%
Platelet	0.30%	Red cell volume distribution width	0.40%
Neutrophils count	25.80%	Lymphocyte count	25.80%
Monocyte count	25.80%		
Aspartate aminotransferase	7.20%	Alanine transaminase	6.90%
Alkaline phosphatase	7.00%	Serum lipase	26.10%
Serum amylase	65.50%	Total bilirubin	7.70%
Direct bilirubin	83.50%	Indirect bilirubin	84.70%
Creatine kinase	67.50%	Creatine kinase isoenzyme	70.70%
Glutamyl transpeptidase	98.60%	Lactate dehydrogenase	39.50%
Anion gap	0.30%	Total protein	97.90%
Albumin	28.00%	Globulin	99.10%
Bicarbonate	0.30%	Blood urea nitrogen	0.40%
Serum creatinine	0.30%	Serum sodium	0.30%
Serum chlorine	0.30%	Serum potassium	0.30%
Serum calcium	1.70%	Blood glucose	0.40%
Triglycerides	27.6	Total cholesterol	63.40%
High-density lipoprotein	66.20%	Low-density lipoprotein	69.40%
Internationalized normalized ratio	7.80%	Prothrombin time	7.80%
D dimer	99.70%	Fibrinogen	71.90%
Thrombin	100%	Activates partial thromboplastin time	9.90%
Lactic acid	51.90%	PH	46.20%
Partial pressure of oxygen	46.20%	Partial pressure of carbon dioxide	46.20%
Base excess	46.20%	Oxygenation index	59.10%
Interventions (treatment or medical operation performed in the first 24 h after admission to the ICU)			
Mechanical ventilation	0%	Renal replacement therapy	0%
Vasoactive drugs are used	0%		
Comorbid disease			
Hypertension	0%	Liver disease	0%
Diabetes	0%	Coagulation dysfunction	0%
Myocardial infarction	0%	Malignant tumor	0%
Congestive heart failure	0%	History of drinking alcohol	0%
Chronic lung disease	0%	Obesity	0%
Atrial fibrillation	0%	Hyperlipidemia	0%
Chronic kidney disease	0%	End stage renal disease	0%
Risk factors (assessed in the first 24 h after admission to the ICU)			
Sepsis	0%	Antibiotics are used	0%
Disease severity score (assessed in the first 24 h after admission to the ICU)			
CCI score	0%	Ranson score	0%
BISAP score	0%	APACHE II score	0%
Outcome measures (occurred within 7 days of admission to the ICU)			
Acute kidney injury (AKI)	0%	AKI stage	0%

ICU refers to intensive care unit, AKI refers to acute kidney injury, CCI refers to Charson comorbidity index, BISAP refers to bedside index for the severity of acute pancreatitis, APACHE II refers to acute physiology and chronic health evaluation II.

## Appendix 2.

Baseline characteristics of the populations studied in two groups, the training cohort and the validation cohort

**Table:**

Variables	Total (*n* = 799)	Training cohort (*n* = 560)	Validation cohort (*n* = 239)
Age, (years)	59.06 (17.40)	59.17 (17.43)	58.81 (17.38)
Male, *n*(%)	458 (57.32%)	337 (60.18%)	121 (50.63%)
Ethnicity, *n*(%)			
White	503 (62.95%)	360 (64.29%)	143 (59.83%)
Black	77 (9.64%)	50 (8.93%)	27 (11.30%)
Other	219 (27.41%)	150 (26.79%)	69 (28.87%)
Comorbid disease, *n*(%)			
Hypertension	494 (61.83%)	360 (64.29%)	134 (56.07%)
Diabetes	241 (30.16%)	180 (32.14%)	61 (25.52%)
Myocardial infarction	77 (9.64%)	52 (9.29%)	25 (10.46%)
Congestive heart failure	146 (18.27%)	108 (19.29%)	38 (15.90%)
Atrial fibrillation	162 (20.28%)	117 (20.89%)	45 (18.83%)
Hyperlipidemia	312 (39.05%)	218 (38.93%)	94 (39.33%)
Chronic lung disease	171 (21.40%)	129 (23.04%)	42 (17.57%)
Chronic kidney disease	92 (11.51%)	62 (11.07%)	30 (12.55%)
Liver disease	232 (29.04%)	165 (29.46%)	67 (28.03%)
Malignant tumor	74 (9.26%)	49 (8.75%)	25 (10.46%)
Obesity	101 (12.64%)	71 (12.68%)	30 (12.55%)
History of drinking alcohol	293 (36.67%)	211 (37.68%)	82 (34.31%)
Vital signs			
Heart rate, (times/min)	100.73 (21.05)	101.73 (21.20)	98.38 (20.53)
Respiratory, (times/min)	21.46 (6.37)	21.67 (6.45)	20.96 (6.14)
Temperature, (°C)	36.86 (0.92)	36.90 (0.89)	36.78 (0.99)
Systolic blood pressure, (mmHg)	127.75 (25.06)	128.49 (24.84)	126.02 (25.53)
Diastolic blood pressure, (mmHg)	74.34 (19.27)	74.56 (18.87)	73.83 (20.20)
Mean arterial pressure, (mmHg)	87.01 (19.38)	87.31 (19.09)	86.29 (20.08)
Oxygen saturation, (%)	96.00 (94.00–99.00)	96.00 (94.00–99.00)	97.00 (94.50–99.00)
Laboratory indicators, (Reference range, Unit)			
WBC, (4–10, ×10^9^/L)	12.80 (8.80–18.20)	12.85 (9.00–18.20)	12.60 (8.35–17.80)
HB, (14–18, g/dL)	11.59 (2.58)	11.58 (2.61)	11.62 (2.54)
HCT, (36–48, %)	35.07 (7.61)	34.99 (7.70)	35.25 (7.39)
PLT, (150–440, ×10^9^/L)	201.00 (140.00–289.50)	202.00 (140.75–293.25)	194.00 (139.00–281.50)
RDW, (10.5–15.5, %)	15.05 (1.99)	15.00 (1.96)	15.17 (2.04)
ALT, (0–40, IU/L)	50.00 (25.00–141.00)	53.00 (24.00–151.00)	45.00 (25.00–117.50)
AST, (0–40, IU/L)	76.00 (35.00–181.50)	77.00 (35.00–179.50)	73.00 (34.50–192.50)
ALP, (35–105, IU/L)	104.00 (69.00–165.50)	106.00 (69.75–169.75)	94.00 (66.00–156.50)
Total bilirubin, (0–1.5, mg/dL)	1.00 (0.50–2.70)	1.00 (0.50–2.90)	1.00 (0.50–2.35)
AG, (10–18, mEq/L)	16.00 (13.00–20.00)	16.00 (13.00–19.25)	16.00 (14.00–20.00)
Bicarbonate, (22–32, mEq/L)	20.87 (5.75)	20.82 (5.84)	20.99 (5.56)
BUN, (6–20, mg/dL)	19.00 (12.00–34.00)	19.00 (12.00–33.00)	20.00 (12.00–35.00)
SCr, (0.5–1.2, mg/dL)	1.00 (0.70–1.70)	1.00 (0.70–1.80)	1.00 (0.70–1.60)
Serum Sodium, (133–145, mg/dL)	137.80 (5.76)	137.82 (5.74)	137.75 (5.84)
Serum chlorine, (96–108, mg/dL)	103.11 (7.55)	103.18 (7.57)	102.92 (7.51)
Serum potassium, (3.3–5.1, mg/dL)	4.22 (0.88)	4.22 (0.89)	4.22 (0.87)
Serum calcium, (8.4–10.3, mg/dL)	7.97 (1.07)	7.92 (1.09)	8.09 (1.02)
Blood glucose, (70–100, mg/dL)	129.00 (104.00–183.00)	127.00 (103.75–178.25)	137.00 (105.00–190.50)
INR, (0.9–1.1)	1.30 (1.10–1.50)	1.30 (1.10–1.50)	1.30 (1.10–1.55)
PT, (9.4–12.5, s)	14.10 (12.70–16.50)	14.20 (12.80–16.42)	13.90 (12.50–17.05)
APTT, (25–36.5, s)	30.00 (26.55–36.45)	29.70 (26.50–35.52)	30.90 (26.60–38.30)
Interventions, *n*(%)			
Mechanical ventilation	316 (39.55%)	218 (38.93%)	98 (41.00%)
RRT	44 (5.51%)	35 (6.25%)	9 (3.77%)
Vasoactive drugs are used	222 (27.78%)	152 (27.14%)	70 (29.29%)
Injury factors, *n*(%)			
Sepsis	525 (65.71%)	370 (66.07%)	155 (64.85%)
Antibiotics are used	521 (65.21%)	372 (66.43%)	149 (62.34%)
Disease severity score, (points)			
CCI score	4.00 (2.00–6.00)	4.00 (2.00–6.00)	4.00 (2.00–6.00)
BISAP score	3.00 (2.00–3.00)	3.00 (2.00–3.00)	3.00 (2.00–3.00)
Ranson score	3.00 (2.00–4.00)	3.00 (2.00–4.00)	3.00 (2.00–4.00)
APACHE II score	20.54 (8.11)	20.62 (8.02)	20.37 (8.34)
Outcome-related measures			
Length of ICU, (days)	3.08 (1.80–7.41)	3.05 (1.80–7.01)	3.13 (1.83–7.92)
Length of hospital, (days)	11.81 (6.63–21.71)	11.68 (6.50–21.81)	12.29 (6.88–20.74)
ICU mortality, *n*(%)	62 (7.76%)	40 (7.14%)	22 (9.21%)
Hospital mortality, *n*(%)	99 (12.39%)	62 (11.07%)	37 (15.48%)
AKI, *n*(%)	499 (62.45%)	346 (61.79%)	153 (64.02%)
AKI stage, *n*(%)			
AKI stage 1	100 (12.52%)	64 (11.43%)	36 (15.06%)
AKI stage 2	212 (26.53%)	151 (26.96%)	61 (25.52%)
AKI stage 3	187 (23.40%)	131 (23.39%)	56 (23.43%)

Note: AKI refers to acute kidney injury, CKD refers to chronic kidney disease, WBC refers to white blood cell count, HB refers to hemoglobin, HCT refers to hematocrit, PLT refers to platelets, RDW refers to red cell volume distribution width, ALT refers to alanine aminotransferase, AST refers to aspartate aminotransferase, ALP refers to alkaline phosphatase, AG refers to anion gap, BUN refers to blood urea nitrogen, SCr refers to serum creatinine, INR refers to international normalized ratio, PT refers to prothrombin time, APTT refers to activating partial thrombin time, RRT refers to renal replacement therapy, CCI refers to charson comorbidity index, BISAP refers to bedside index for the severity in acute pancreatitis, APACHE II refers to acute physiology and chronic health evaluation II, ICU refers to intensive care unit; 1 kPa ≈ 7.5 mmHg.
